# Artificial Intelligence May Predict Early Sepsis After Liver Transplantation

**DOI:** 10.3389/fphys.2021.692667

**Published:** 2021-09-06

**Authors:** Rishikesan Kamaleswaran, Sanjaya K. Sataphaty, Valeria R. Mas, James D. Eason, Daniel G. Maluf

**Affiliations:** ^1^Emory University School of Medicine, Atlanta, GA, United States; ^2^Georgia Institute of Technology, Atlanta, GA, United States; ^3^Sandra Atlas Bass Center for Liver Diseases & Transplantation, Northshore University Hospital, Northwell Health, Manhasset, NY, United States; ^4^University of Maryland School of Medicine, Baltimore, MD, United States; ^5^Transplant Institute, University of Tennessee, Memphis, TN, United States

**Keywords:** machine learning, liver transplant, surgery, physiological data streams, artificial intelligence, sepsis

## Abstract

**Background:** Sepsis, post-liver transplantation, is a frequent challenge that impacts patient outcomes. We aimed to develop an artificial intelligence method to predict the onset of post-operative sepsis earlier.

**Methods:** This pilot study aimed to identify “physiomarkers” in continuous minute-by-minute physiologic data streams, such as heart rate, respiratory rate, oxygen saturation (SpO2), and blood pressure, to predict the onset of sepsis. The model was derived from a cohort of 5,748 transplant and non-transplant patients across intensive care units (ICUs) over 36 months, with 92 post-liver transplant patients who developed sepsis.

**Results:** Using an alert timestamp generated with the Third International Consensus Definition of Sepsis (Sepsis-3) definition as a reference point, we studied up to 24 h of continuous physiologic data prior to the event, totaling to 8.35 million data points. One hundred fifty-five features were generated using signal processing and statistical methods. Feature selection identified 52 highly ranked features, many of which included blood pressures. An eXtreme Gradient Boost (XGB) classifier was then trained on the ranked features by 5-fold cross validation on all patients (*n* = 5,748). We identified that the average sensitivity, specificity, positive predictive value (PPV), and area under the receiver-operator curve (AUC) of the model after 100 iterations was 0.94 ± 0.02, 0.9 ± 0.02, 0.89 ± 0.01, respectively, and 0.97 ± 0.01 for predicting sepsis 12 h before meeting criteria.

**Conclusion:** The data suggest that machine learning/deep learning can be applied to continuous streaming data in the transplant ICU to monitor patients and possibly predict sepsis.

## Introduction

Liver transplantation continues to be the optimal and more successful therapy for end-stage liver disease and cirrhosis (Kim et al., 2019). One of the biggest challenges in the transplant community is the discrepancy of donor availability and the need of the recipients. Transplant centers frequently appeal to the use of marginal or suboptimal donors to decrease this gap, while, at the same time, increasing the chances for post-transplant complications associated with organ dysfunction (Kim et al., 2019).

Moreover, in recent years, the increased availability of more potent immunosuppressive agents, along with sicker and older recipients needing transplantation, has increased the incidence of opportunistic infections (OIs) affecting patient survival after liver transplantation (LT) (Haidar et al., 2019; He et al., 2019). Post-transplant infections with or without surgical complications are the leading cause of morbidity and mortality post-LT (Kim et al., 2019). Overall, infections and sepsis are estimated to occur in more than half of LT recipients, and are the main cause of post-LT death between days 21 and 180 (Sun et al., 2011; Fischer et al., 2013; Martin et al., 2014; Haidar et al., 2019; He et al., 2019). Bacterial infections are the most common post-transplant infections (>70%), followed by viral and fungal infections (Sun et al., 2011; Haidar et al., 2019; He et al., 2019). Fortunately, due to intensive screening practices to detect latent infections in liver transplant candidates, and with the implementation of appropriate prophylactic protocols and therapy, mortality associated with post-LT infections is still low (<10%) (Sun et al., 2011; Martin et al., 2014; He et al., 2019). Known risk factors associated with infection after LT include a high model for end-stage liver disease (MELD) score, re-transplantation, advanced age of the recipient, number of blood transfusions, renal replacement therapy (RRT), and a long intensive care unit (ICU) stay, among others (Haidar et al., 2019; He et al., 2019). Several steps in the physical examination and laboratory assessment allow a clinician to identify active infections that would prompt therapy to prevent complications. It is known, however, that delays in diagnosis and therapy implementations would carry higher mortality in this population (Kumar et al., 2006; Dombrovskiy et al., 2007). Because of the scarce resource of liver grafts and the associated mortality of post-transplant infections, biomarkers or markers capable of accurately expediting diagnosis would be of significant clinical significance in a transplant unit.

Sepsis is a common event, with more than a million Americans getting hospitalized each year (Dombrovskiy et al., 2007; Liu et al., 2014). Sepsis is caused by a heightened inflammatory response to an infection, and can quickly progress to multi-organ failure and death (Liu et al., 2014). In the septic shock phase of the disease, every hour that treatment is delayed can lead to a 7.6% increase in mortality (Kumar et al., 2006). In liver transplantation, this phenomenon is not different in the general population, and an early infection due to surgical complications, such as bleeding, bile leak, or rejection, may trigger infections and sepsis with severe consequences in recipients (Kumar et al., 2006; Elkholy et al., 2019).

A number of recent studies have applied artificial intelligence (AI) and machine learning to identify patients at risk for sepsis earlier, thereby potentially reducing mortality and morbidity (Kumar et al., 2006; Nemati et al., 2018; Elkholy et al., 2019). These methods have typically used an array of clinical and laboratory variables in the electronic medical record (EMR) to predict the risk of sepsis. While such methods have achieved a significant performance in retrospective studies, they are limited by the aperiodic and unstructured nature of EMR data. Alternative methods for developing predictive models for sepsis have used high-frequency data streams captured from the medical monitor, such as heart rate, blood pressures, respiratory rate, and oxygen saturation (Kamaleswaran et al., 2018; van Wyk et al., 2019). The use of such biosensor data may identify physiomarkers that present hours before the clinical manifestation of the disease or event, thereby allowing for earlier recognition and the initiation of therapy. In this study, we evaluated the effectiveness of high-frequency physiological data stream analysis in predicting the onset of sepsis in liver transplant patients. We developed and tested a number of machine learning methods using features derived from the physiological time series to generate predictions at various time intervals before the Third International Consensus Definition of Sepsis (Sepsis-3) clinical definition (Singer et al., 2016).

## Materials and Methods

### Data Collection Environment

This observational retrospective study was approved by the Institutional Review Board (IRB) of the University of Tennessee Health Science Center. We collected continuous physiological data streams from bed-side monitors using the Cerner iBus (Cerner Corporation, Kansas City, MO, United States) (Cerner Corporation, 2014). The Cerner iBus generated minute-by-minute heart rate (HR), respiratory rate (RR), blood pressure (mean, systolic, and diastolic), and oxygen saturation (SpO_2_) data streams; however, continuous temperature was not available and was, therefore, excluded from the analysis. We captured non-invasive blood pressure (NIBP), which was sampled at least once an hour, and, in some cases where clinical deterioration was suspected, the NIBP was sampled more frequently.

### Case Definition

Patients admitted to the intensive care unit across the Methodist University Hospital and Transplant Institute (UTHSC) between January 2017 and January 2020, with continuous minute-by-minute physiological monitoring data, were included in the study. In this study, we utilized the Sepsis-3 definition [SHAP (SHapley Additive exPlanations), 2021]; patients who met Sepsis-3 criteria but did not have high-frequency data recorded within the prior 24 h were excluded. Sepsis-3 definitions were applied serially using the method described by Nemati et al. (2018) in order to identify the time of sepsis onset (event time) (Nemati et al., 2018). We identified controls as those who had never met sepsis criteria during their encounter. To identify a control event time (for supervised learning), we used a randomly generated timestamp that occurred between admission and discharge, provided that the 24-h data availability criterion prior to the random event time was met. All the data were then temporally aligned to the event time, identified as t_sepsis_

### Feature Extraction and Feature Selection

For each of the six physiological data streams [heart rate, respiratory rate, oxygen saturation, systolic blood pressure (SBP), diastolic blood pressure (DBP), and mean femoral artery blood pressure (MAP)], features were extracted using eight statistical and two time-frequency domain methods, namely, mean, sum, minimum, maximum, frequency of the measurement (length), standard deviation, variance, kurtosis, fast Fourier transform (FFT), and continuous wavelet transform (CWT) (Christ et al., 2018). A number of parameters were included for evaluating the FFT coefficients (0–100, with a step of 4); then, we extracted the absolute coefficient values for each parameter. For CWT features, we evaluated width values of 0–20 at a step of 2. These features were extracted for each hour within the 3-h window across six data streams, for a total of 774 features per window; 24 FFT features consistently returned null and subsequently removed, resulting in a total of 750. Missing data were imputed if there was a previous record; otherwise, we used the population median value. These features were then concatenated into a single feature vector that incorporated temporal dynamics over the 3-h period.

We then applied a variety of feature selection methods, including statistical, and thus performed both non-parametric Mann–Whitney-U and parametric independent sample *t*-tests, ridge, lasso, recursive feature elimination (RFE), and random forest-based variable importance utilizing information gain and gini impurity. These feature selection methods were performed in order to reduce data dimensionality to a limited set of markers that predict the onset of sepsis.

The dataset was then segmented into two cohorts; the first included all patients who were admitted to the intensive care unit without having received a liver transplant at least 31 days prior to admission, and the second cohort included all patients who underwent transplantation. For the training of the model, we implemented a subsampling strategy where we randomly selected an equal number of controls to cases. In order to control for over-fitting, we implemented a 5-fold cross validation on each iteration to derive training and test performances. We then iterated this training 100 times to generate unique model performances from each run and reported the averaged performance measure overall runs. Hyperparameters were evaluated using a grid-search approach, with which we predefined the upper and lower limits of the hyperparameters and generated a series of models and recorded their performance. The hyperparameters that achieved the most stable model performance, with minimal variance over the 100 runs, were selected and used to train the entire first cohort data. We selected the optimal hyperparameter for each of the algorithms that were explored, namely, eXtreme Gradient Boosting (XGB), logistic regression (LR), support vector machine (SVM), and random forest (RF). The remaining selected models were then validated on the transplant cohort.

### Machine Learning Pipeline

Prior to the modeling of high-dimensional data streams, we applied an unsupervised cluster visualization technique called t-distributed stochastic neighbor embedding (tSNE) (Van der Maaten and Hinton, 2008). This method converts similarities between data points to joint probabilities and tries to minimize the divergence between these joint probabilities in a low-dimensional manner to illustrate possible clusters and separation. Then, in the binary classification, we applied a number of machine learning classifiers to generate complementary but competing models. We investigated supervised learning methods such as eXtreme Gradient XGB, LR, SVM, and RF, with both XGB and RF being non-linear ensemble-based learning methods. In particular, XGB is unique in incorporating sequential boosting to improve classification performance, but it may also be sensitive to overfitting. Furthermore, SVM is a classical machine learning method that utilizes hyperplanes to optimize separation among features and has been successfully used for binary classification tasks. In addition, ALR is a statistical learning method and often serves as a benchmark for machine learning model comparison. We utilized the above algorithms to compare performance across unique learning strategies to select an optimal algorithm that performs best for this dataset.

In order to generate explainable feature importance, we used the SHapley Additive exPlanations (SHAP) package (Lundberg and Lee, 2017). The SHAP algorithm uses methods from game theory to explain the output of machine learning models; it has been noted to be state-of-the-art in terms of generating reliable explanations of predictive model outputs.

Model benchmarks were generated by computing area under the receiver-operator curve (AUC), area under the precision-recall curve (AUPRC), sensitivity, specificity, and positive predictive value (PPV). In particular, AUC is a traditional benchmarking tool for determining performance over a range of possible model-estimated probability thresholds; however, it assumes a balanced distribution of samples. Conversely, AUPRC is more useful for measuring performance across imbalanced and low-PPV scenarios; a higher AUPRC indicates that the model can accurately identify all positive examples without compromising specificity.

We utilized Python 3.6 and the XGBoost package (XGBoost Documentation, 2021) for developing the XGB model and the sci-kit learn (Scikit-Learn: Machine Learning in Python, 2021) package for developing the remaining machine learning and statistical analysis code base. We utilized the SHAP library to derive explainable interpretations and summary plots [SHAP (SHapley Additive exPlanations), 2021].

## Results

### Data Missingness

In the derivation dataset, the rate of missing value was highest between MAP and DBP, with an average of 16% patients having at least one missing value in the 3-h observational window. Oxygen saturation was the most often recorded, with only 0.1% of the patients missing this measure, followed by HR with a missing value of up to 0.6%, RR in 2.7% of patients, and SBP in 4.8%. Supplementary Figure S1 illustrates the correlation between the missing variables, and suggests that when MAP is missing, DBP is also missing and vice versa. In 60% of the cases, SBP is associated with missing MAP and DBP. In cases where HR is missing, in 30% of the patients, MAP and DBP are also missing.

We identified a total of 5,748 non-transplant patients who were admitted to the intensive care unit over an 8-month period, 604 (10.5%) of whom met the “Sepsis-3” criteria defined as suspicion of infection in the presence of organ failure (Singer et al., 2016). Furthermore, another 252 patients were separately identified to have undergone a liver transplant, 92 (36%) of whom met Sepsis-3 criteria during their stay in the ICU.

Age and gender differences were not statistically significant in the general cohort (Table 1). Model for end-stage liver disease scores was also not statistically different between the cohorts, with scores consistently ranging from 22 to 28 across both cohorts. As expected, in the transplant program, a greater portion of the transplant cohort consisted of male Caucasians. In-hospital mortality in the transplantation cohort was 9%, which is less than the in-hospital mortality in the general cohort. The incidence of sepsis in the transplant cohort was significantly higher than in the general cohort. The median age of the transplant cohort was 57 years, with the sepsis patients being, on average, 4 years older than the non-sepsis liver transplant patients across each group similar to the general cohort. All patients in the transplant cohort were temporarily mechanically ventilated, while only 23% was in the general cohort.

**Table 1:** Characteristics of the study population.

	**Sepsis (Non-Transplant)**			**Sepsis (Transplant)**		
**Characteristics**	**Overall**	**Yes**	**No**	**Overall**	**Yes**	**No**
Patient, n (%)	5,748 (100)	604 (10.5)	5,144 (89.5)	252 (100)	92 (27)	160 (73)
Male, n (%)	2,932 (49.2)	299 (48.5)	2,633 (49.3)	160 (63)	50 (54)	110 (69)
Mechanical ventilation, n (%)	1,356 (22.8)	490 (74.6)	896 (16.8)	252 (100)	92 (100)	160 (100)
In hospital deaths, n (%)	439 (7.4)	176 (28.5)^**^	263 (4.9)	23 (9)	8 (9)	15 (9)
Age (yr.) median (IQR)	62 (50–72)	63 (52–72)	61.5 (50–72)	57 (48–66)	61 (46–67)	57 (50–65)
ICU LOS (d), median (IQR)	5 (3–8)	11 (6–20)^**^	4 (3–7)	2 (2–5)	4 (2–5)	2 (2–5)
ICU LOS >= 7d, n (%)	1,917 (32.2)	435 (70.5)^**^	1,482 (27.7)	58 (22.9)	21 (23)	37 (22.9)
Self-reported race, n (% row-wise)						
Black or African American	3,453 (58.0)	387 (62.7)^*^	3,066 (57.4)	44 (17)	7 (16)	37 (84)
White	2,360 (39.6)	214 (34.7)	2,146 (40.2)	178 (71)	14 (8)	164 (80)
Other/Unknown	104 (1.8)	13 (2.1)	91 (1.7)	20 (8)	2	18 (6)
Multiple	21 (0.4)	1 (0.2)	20 (0.4)	0	0	0
Asian	19 (0.3)	2 (0.3)	17 (0.3)	1	0	1
Self-reported Ethnicity, n (%)						
Not Hispanic or Latino	5,847 (98.2)	605 (98.0)	5,242 (98.2)	219 (87)	11 (1)	208 (99)
Hispanic or Latino	69 (1.2)	8 (1.4)	69 (1.3)	19 (8)	2 (10)	17 (90)
Unknown or Declined	33 (0.6)	4 (0.6)	29 (0.5)	0	0	0

* *Significant at a = 0.01*;

** *significant at a = 0.001*.

**Figure 1:**
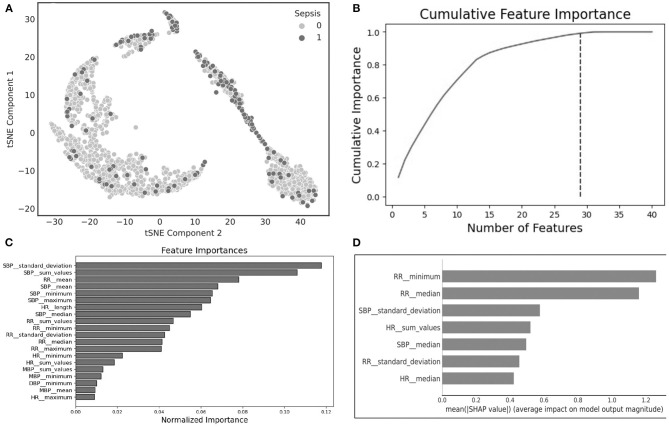
**(A)** t-Distributed stochastic neighbor embedding (tSNE) plot characterizing sepsis (dark purple) and non-sepsis clusters (pink); **(B)** cumulative feature importance identifies 29 features that explain 99% of the data; **(C)** feature importance generated using light gradient boosted machines; **(D)** explainable importance with SHapley Additive exPlanations (SHAP) after feature selection.

An Unsupervised clustering, using the tSNE method, of the raw data up to 12 h prior to sepsis onset suggests that clusters can be distinguishable (Figure 1A, tSNE plot). The cluster to the left largely consists of patients without sepsis, while the cluster to the top and to the right contains a significant portion of patients with sepsis, indicating that further analysis of the data may reveal useful predictive markers for sepsis. We found a number of overlapping distinguishing physiomarkers when we utilized the gradient boosting method (Figures 1B,C), and the SHAP output (Figure 1D). Notably, HR, RR, and SBPs were significant explainers for patients who developed sepsis early in the clinical course.

**Figure 2:**
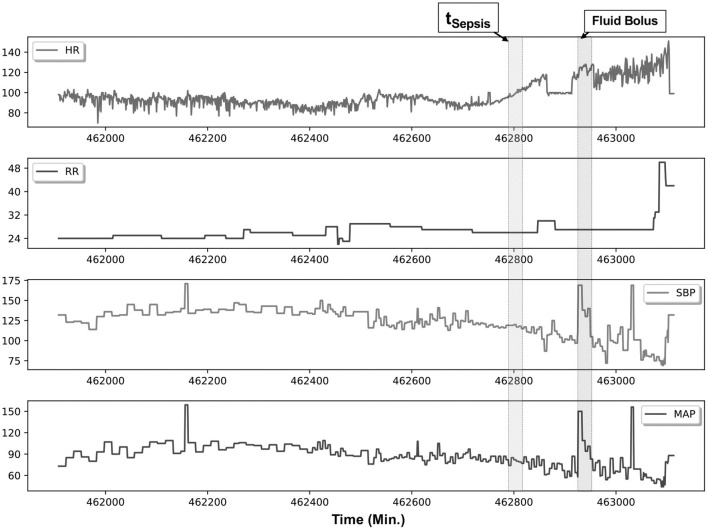
An example patient with sepsis is illustrated in this figure; continuous physiological data were captured over an 18-h post-transplantation period. The patient met Sepsis-3 criteria (t_Sepsis_) 13 h post transplantation (retrospectively identified), and fluid resuscitation (fluid bolus) was initiated 1.5 h thereafter. Several elements are of note within this patient, namely, in the preceding hours before meeting criteria, heart rate (HR) variability is noticeably reduced, accompanied by increased dynamics in the systolic blood pressure (SBP) and mean femoral artery blood pressure (MAP) data streams.

Figure 2 illustrates an example patient with sepsis where the physiological data streams were available up to 16 h before onset. In this figure, dynamic shifts are seen in the HR, RR, and blood pressure data streams during the time leading to sepsis. Moreover, interventional response *via* fluid resuscitation is also observed shortly thereafter.

### Statistical Analysis

**Figure 3:**
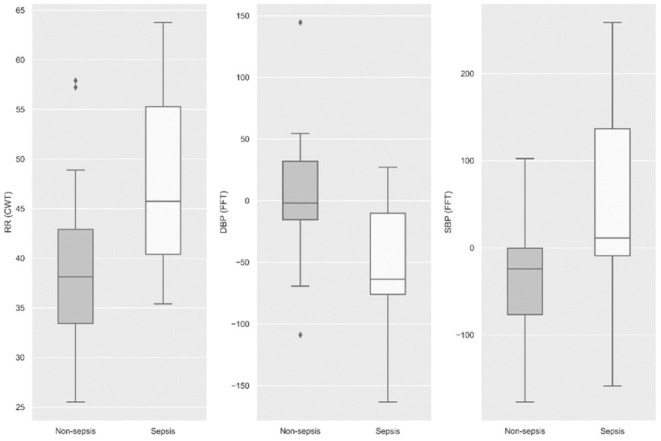
Statistical significance (*p* < 0.001) was observed among the three physiological signals, namely, respiratory rate, diastolic blood pressure, and systolic blood pressure at 12 h prior to sepsis onset.

A total of 750 features were generated from all the physiological data streams using statistical and time-frequency domain methods (described in the methods section); these represent features generated in the observational window at 12 h (prediction horizon) prior to sepsis onset. By Student's *t*-test against these continuous measures to identify distinguishing features, we found that the statistical significance for the transplant cohort at *p* < 0.05 was observed in 311 features, of which 106 were various time-frequency abstractions of DBP and 79 features were related to SBP, 73 to RR, 38 to MAP, and 15 belonged to HR. None of the SpO_2_ features figured as statistically significant. Among the signal processing features, at *p* < 0.001, FFT of DBP and SBP, and CWT of RR were significant (Figure 3). The box plots illustrated in Figure 3 show that frequency-domain characteristics were meaningfully distinguishable among blood pressures, while more complex dynamics that spanned the time-frequency domain were apparent among respiratory rates.

### Machine Learning

**Figure 4:**
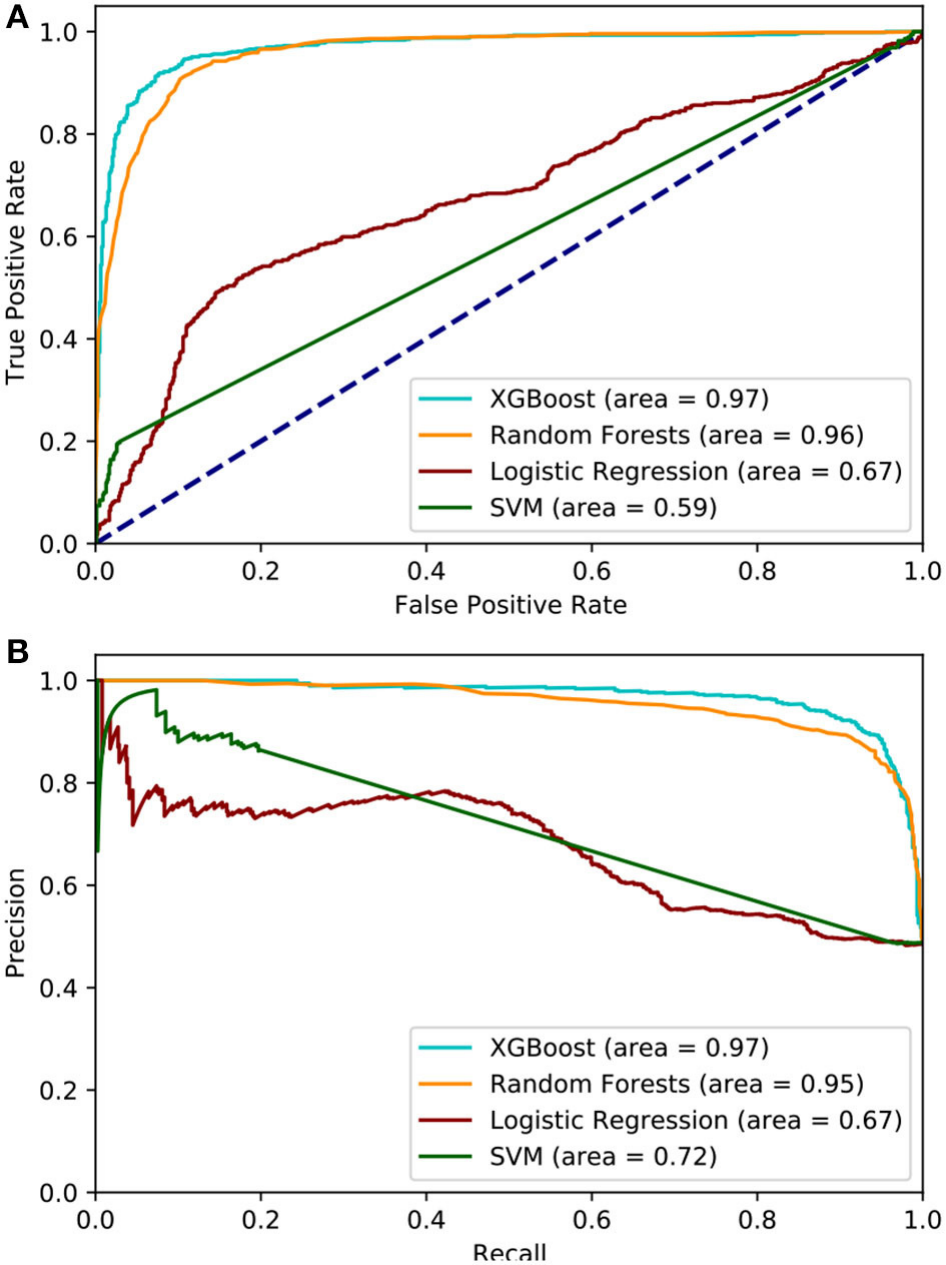
**(A)** Area under the Receiver Operator Curve, the XGBoost model achieves the highest AUC while SVM performed the worst. **(B)** area under the Precision Recall curve, XGBoost maintains the best AUPRC but Logistic Regression is ranked the worst.

Utilizing the statistically significant features (*n* = 311), we applied feature selection techniques, namely, the RFE method, which generated 22 features that were highly predictive. All of these 22 features were derived from statistical and continuous wavelet transform methods, and indicated that SBP characteristics are the top predictor of sepsis (Figure 1D). Separately, using ridge and lasso feature selection, we applied a defined coefficient threshold of 0.5 to select the most predictive features. The lasso method selected 12 features, which consisted exclusively of statistics from respiratory rate. The ridge method selected 52 highly ranked features, of which the top feature was SBP, with various temporal permutations of SBP appearing a total of nine times. The second most important feature was DBP, which appeared a total of 10 times, followed by RR, which appeared 12 times. The models were developed using both the RFE and ridge methods, and the ridge-based feature selection was identified as the optimal feature set because of its improved performance across the 5-fold cross-validation benchmarks. While we evaluated XGB, LR, SVM, RF, and MLP, XGB was identified as the optimal model after averaging 10 randomized runs of the 5-fold cross-validation. Because of the significant overfitting that occurred in the MLP pipeline early in the analysis, we did not pursue it for further hyperparameterization. Figure 4 illustrates model performance, such as AUC and AUPRC, for the machine learning methods evaluated. In the figure, both XGB and RF are consistently shown to have the highest performance across both benchmarks, with XGB slightly outperforming RF.

Table 2 lists the performances of the logistic regression, support vector machine, random forest, and eXtreme Gradient Boost models. The XGB model was identified as the optimal model because of generally improved performance across all metrics, with a mean sensitivity of 0.94, specificity of 0.90, and an AUC of 0.97, as shown in Figure 4. The SVM model performed worst with respect to AUC (0.63) but had the highest specificity (0.94). The RF model performed relatively close to the XGB model but with a lower sensitivity (0.92) and specificity (0.88). The LR model had the lowest overall PPV (0.76). The XGB model outperformed all the other models in terms of each metric except for specificity. The optimal hyperparameters used in the XGB model were as follows: max depth of 6, subsample parameter of 1, the minimum sum of instance weight for child of 1, and a learning rate of 0.1. The optimal SVM kernel function was linear. The threshold used for binary classification was 0.5.

**Table 2:** Comparison of model performance.

	**LR**	**SVM**	**RF**	**XGB**
	**mean [95% CI]**			
Sensitivity	0.49 [0.44–0.49]	0.28 [0.27–0.33]	0.92 [0.90–0.92]	**0.94 [0.93**–**0.95]**
Specificity	0.85 [0.83–0.86]	0.94 [0.93–0.95]	0.88 [0.83–0.89]	**0.90 [0.89**–**0.90]**
PPV	0.76 [0.74–0.78]	0.83 [0.82–0.84]	0.88 [0.87–0.89]	**0.89 [0.88**–**0.91]**
AUC	0.67 [0.66–0.70]	0.63 [0.62–0.65]	0.96[0.93–0.96]	**0.97 [0.95**–**0.97]**

## Discussion

Liver transplantation is a life-saving therapy for patients with liver cancer and end-stage liver disease. In the United States in 2017, more than 7,000 LTs were performed (Kim et al., 2019).

Transplant recipients are, however, at high risk for complications such as infections due to advanced age, obesity, comorbidities, and issues associated with the transplant event that may be related to surgical complications or organ dysfunction (Pedersen and Seetharam, 2014). Furthermore, systemic immunosuppression has rendered liver recipients susceptible to *de novo* infections and the reactivation of preexisting latent infections such as viral infections. Infections occurring during the first month post-LT are usually nosocomial or donor-derived or the result of a perioperative complication, such as a surgical complication, or organ dysfunction (Hernandez Mdel et al., 2015). A recent review of the Organ Procurement and Transplantation Network (OPTN) data from 64,977 patients who underwent liver transplantation identified the incidence of 90-day and 1-year mortalities at 5 and 10%, respectively. Although death associated with cardiovascular/cerebrovascular/pulmonary/hemorrhage was the most common cause of death within the first 21 days (7-day: 53%), only 20% of patients who underwent liver transplantation died from these causes after 180 days. Importantly, infections were the most frequent cause of death 30–180 days after liver transplantation. In contrast, after roughly 200 days from the time of liver transplantation, other causes were the most frequent cause of death (Baganate et al., 2018).

Severe sepsis, or infection with systemic inflammation, poses a substantial burden on the United States healthcare system, leading to >7,50,000 hospitalizations and 2,00,000 deaths annually (Moore et al., 2016). Severe sepsis remains a leading cause of death in the United States, with in-hospital mortality ranging from 12 to 26% (Donnelly et al., 2016). In solid organ transplantation and in contrast to the general belief, infection, and sepsis are more frequent in the general population, but the mortality associated with sepsis is lower, as also demonstrated in the analysis and results (Donnelly et al., 2016).

A big challenge is, however, early diagnosis, as the syndrome of sepsis has a wider range of causative organisms and differing presentations among immunosuppressed individuals such as patients who underwent liver transplant (Oriol et al., 2015). Furthermore, in the septic shock phase of the disease, every hour that treatment is delayed can lead to a 7.6% increase in mortality (Kumar et al., 2006).

Traditional markers of systemic inflammatory response syndrome and clinical presentation may not be present among the immunosuppressed, despite active overwhelming infection (Gauer, 2013).

Hereby, the analysis of patients who underwent liver transplantation patients who were admitted to the intensive care unit post-surgery revealed novel physiomarkers that can predict the onset of sepsis earlier and may have an impact on clinical decisions. An illustration using the tSNE visualization method indicated that there are unique clusters that emerge with separation between sepsis and non-sepsis cohorts. This indicates that the source data, comprising physiological data streams, may indeed be useful to predict the onset of sepsis within this cohort. We further found that these physiomarkers existed at least 12 h before a clinical definition was made. Among the important features, we noted that, when compared across two different explainability methods, we saw a consistent trend in the statistical markers of RR and SBP, along with HR, dominating the list of signals that predicted sepsis early in the clinical course. These vital sign measures have been previously described using EMR data. However, they have not been discussed in the context of continuous bedside monitoring for patients who received liver transplants in the past (Desautels et al., 2016; Bloch et al., 2019). While signal processing methods, FFT and CWT, were both statistically significant between the cohorts, they were outranked by the statistical features derived from the same physiological data streams.

We also found that, while several models may be useful as optimal candidates, the eXtreme Gradient Boost model specifically showed higher performance. In the selection criteria for the optimal model, we ensured that a specificity value of at least 0.6 would be required, as to not overwhelm nursing staff with false alarms. Therefore, these results indicate a value in the use of bedside monitoring data streams for informed clinical decision-making and potential treatment plans for patients who received liver transplants in the past, and may serve as useful alternatives to existing clinical monitoring.

Specific features derived from time-frequency domain extractions revealed the useful characteristics of the continuous physiological data streams that can highly predict sepsis. Specifically, in the results, we found that SBP and DBP, along with changes in RR, were among the dominant features (top 10) in the model. We noted that SpO_2_ was not observed to be a significant predictor. While significant literature has been proposed around the utility of HR and HR variability (Ahmad et al., 2009), we noted that, in the model, these appear only seven times in the 52 features that were included in model development.

We sought to develop a minimal physiologic model of sepsis because of the unpredictable nature of orders and their results. The development, therefore, of a minimalistic predictive model may allow for wider use. However, we note that there may be significant improvements in the performance of the model by incorporating clinical- and laboratory-based findings. We expect this to improve the model performance in prospective deployment.

Limitations associated with this study are being derived from a single site and incomplete data analysis due to incomplete clinical data collection (e.g., IMS, surgical complications, HAT, among others). As for the future study, we seek to incorporate data across multiple sites. We were unable to compute standard severity of illness scores because of the limited clinical data, such as the composite sepsis risk score, D-MELD (donor age recipient MELD), donor risk index, Euro-transplant donor risk index, or survival outcome following liver transplantation (SOFT) score, to perform benchmark comparisons. We also reported a small sample size of patients who received liver transplants in the past, which could limit the generalizability of the model; thus, larger datasets from multi-site transplantation units could improve the external generalization.

### Clinical Translation and Future Study

In this pilot study, we demonstrated that continuous physiological data streams can be used for informed clinical decision-making related to the risk of sepsis among patients who received liver transplants in the past. While the model proposed in this study can be directly applied, clinical translation has been a major challenge for machine learning algorithms. We have previously demonstrated that, while clinical data may be useful by themselves, machine learning algorithms are also influenced by measurement indicators (e.g., practice patterns), such as specific applications of sepsis bundles that may indicate increased clinical suspicion (Futoma et al., 2021). In order to control for these confounding variables, a clinical translation of such machine learning models needs to be carefully managed, for instance, by enacting benchmark methods that include silent prospective pilots and clinical adjudication of alerts. These efforts form the basis for the future study.

## Conclusion

Artificial intelligence is becoming an important tool to assist many areas in the field, such as inpatient and outpatient monitoring, including the setting of solid organ transplantation (Woldaregay et al., 2019). In this context, this is one of the first studies that aim to demonstrate that the use of machine learning and AI tools may accurately assess a large amount of continuous data streams from the bedside of patients and help to make earlier diagnoses or event recognition, allowing for faster and more accurate clinical decisions.

## Data Availability Statement

The data analyzed in this study is subject to the following licenses/restrictions: Restricted due to institutional IRB policy. Requests to access these datasets should be directed to rkamaleswaran@emory.edu.

## Ethics Statement

The studies involving human participants were reviewed and approved by University of Tennessee Health Science Center. Written informed consent for participation was not required for this study in accordance with the national legislation and the institutional requirements.

## Author Contributions

RK and DM participated in research design. DM, RK, and VM participated in the writing of the article. RK, DM, VM, and SS participated in data analysis. RK, DM, and JE participated in performing the research. All authors contributed to the article and approved the submitted version.

## Conflict of Interest

The authors declare that the research was conducted in the absence of any commercial or financial relationships that could be construed as a potential conflict of interest.

## Publisher's Note

All claims expressed in this article are solely those of the authors and do not necessarily represent those of their affiliated organizations, or those of the publisher, the editors and the reviewers. Any product that may be evaluated in this article, or claim that may be made by its manufacturer, is not guaranteed or endorsed by the publisher.
