# Supplementary figures and images for: Artificial Intelligence May Predict Early Sepsis After Liver Transplantation
Source: Front Physiol. 2021 Sep 6;12:692667. doi: 10.3389/fphys.2021.692667 (PMC8450439; doi:10.3389/fphys.2021.692667)

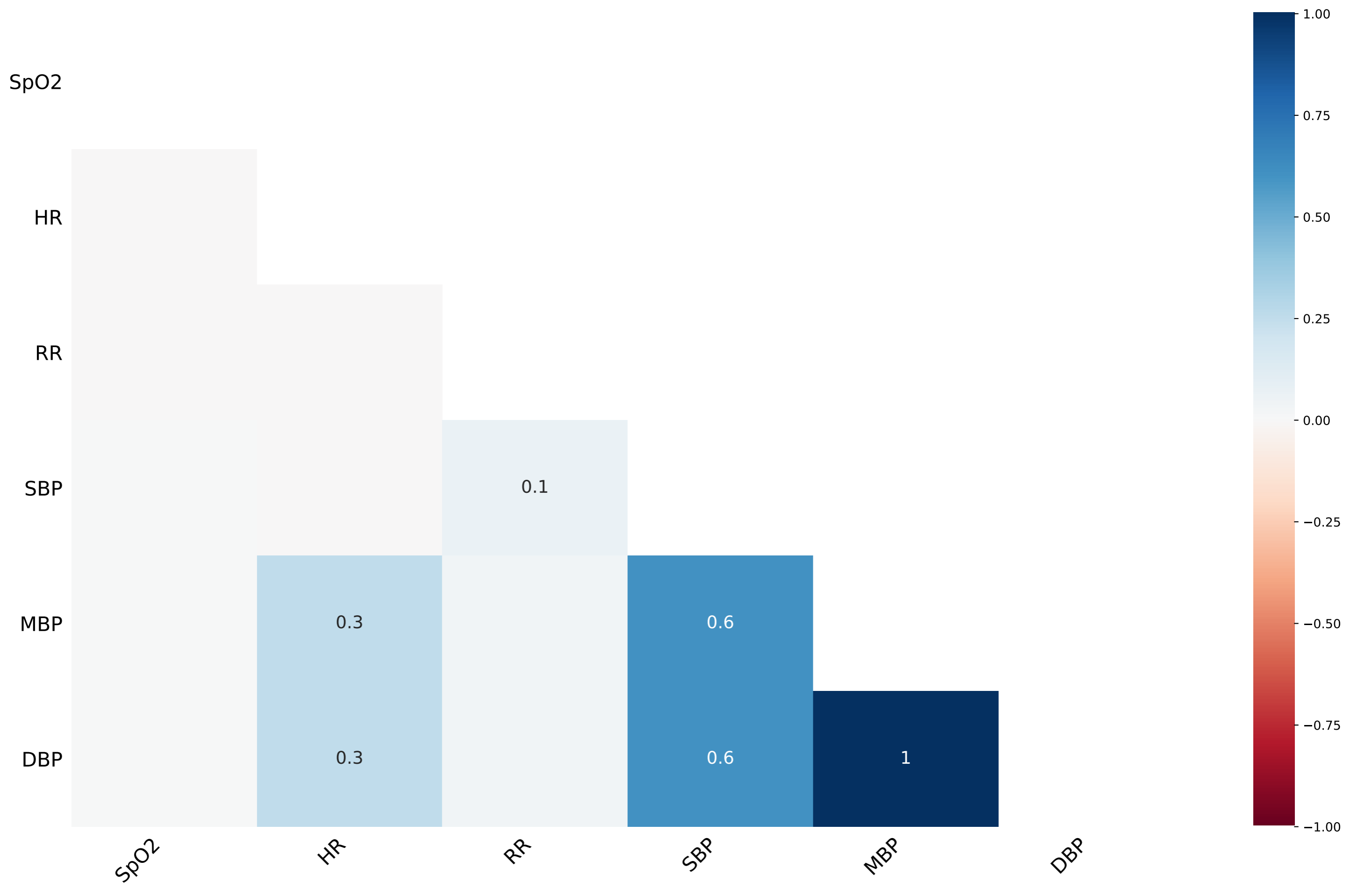

Supplement: Supplementary file 1 [file Image_1.pdf]
